# Development and clinical validation of deep learning for auto-diagnosis of supraspinatus tears

**DOI:** 10.1186/s13018-023-03909-z

**Published:** 2023-06-13

**Authors:** Deming Guo, Xiaoning Liu, Dawei Wang, Xiongfeng Tang, Yanguo Qin

**Affiliations:** 1 Orthopaedic Medical Center, The Second Hospital of Jilin University, Changchun, 130041 People’s Republic of China; 2 Beijing Infervision Technology Co Ltd, Beijing, People’s Republic of China; 3 Jilin Provincial Key Laboratory of Orhtopeadics, Changchun, People’s Republic of China

**Keywords:** Supraspinatus tears, Convolutional neural network, Two-dimensional model, Diagnostic performance and efficiency

## Abstract

**Background:**

Accurately diagnosing supraspinatus tears based on magnetic resonance imaging (MRI) is challenging and time-combusting due to the experience level variability of the musculoskeletal radiologists and orthopedic surgeons. We developed a deep learning-based model for automatically diagnosing supraspinatus tears (STs) using shoulder MRI and validated its feasibility in clinical practice.

**Materials and methods:**

A total of 701 shoulder MRI data (2804 images) were retrospectively collected for model training and internal test. An additional 69 shoulder MRIs (276 images) were collected from patients who underwent shoulder arthroplasty and constituted the surgery test set for clinical validation. Two advanced convolutional neural networks (CNN) based on Xception were trained and optimized to detect STs. The diagnostic performance of the CNN was evaluated according to its sensitivity, specificity, precision, accuracy, and F1 score. Subgroup analyses were performed to verify its robustness, and we also compared the CNN’s performance with that of 4 radiologists and 4 orthopedic surgeons on the surgery and internal test sets.

**Results:**

Optimal diagnostic performance was achieved on the 2D model, from which F1-scores of 0.824 and 0.75, and areas under the ROC curves of 0.921 (95% confidence interval, 0.841–1.000) and 0.882 (0.817–0.947) were observed on the surgery and internal test sets. For the subgroup analysis, the 2D CNN model demonstrated a sensitivity of 0.33–1.000 and 0.625–1.000 for different degrees of tears on the surgery and internal test sets, and there was no significant performance difference between 1.5 and 3.0 T data. Compared with eight clinicians, the 2D CNN model exhibited better diagnostic performance than the junior clinicians and was equivalent to senior clinicians.

**Conclusions:**

The proposed 2D CNN model realized the adequate and efficient automatic diagnoses of STs, which achieved a comparable performance of junior musculoskeletal radiologists and orthopedic surgeons. It might be conducive to assisting poor-experienced radiologists, especially in community scenarios lacking consulting experts.

**Supplementary Information:**

The online version contains supplementary material available at 10.1186/s13018-023-03909-z.

## Introduction

Rotator cuff tears (RCTs) are the most common reason for shoulder pain [1]. An epidemiologic study reported that the prevalence of RCTs was 22.1% in a village in Japan [2]. The most common manifestations are shoulder joint pain and functional impairment, which are difficult to distinguish from frozen shoulder or other diseases [3, 4]. Most RCTs affect the supraspinatus tendon. Previous studies have shown that supraspinatus tear (ST) severity at diagnosis is positively related to the prognosis of the treatment [5]. Furthermore, the partial-thickness tear can quickly develop into severe full-thickness tears. Thus, timely diagnoses and treatment of STs will significantly improve the patients' prognoses [6, 7].

In current clinical practice, most ST diagnoses are based on MRI by a musculoskeletal (MSK) radiologist or orthopedist [8]. However, making an accurate diagnosis may still be challenging for a non-MSK radiologist, a trainee on call, or an orthopedist in a rural area with limited MRI or RCT experience [9]. In primary medical institutions, an auxiliary diagnostic tool to assist radiologists in diagnosing ST is urgently needed.

As an emerging new technique, convolutional neural networks (CNNs) have been increasingly used for medical imaging tasks in recent years, including tissue and lesion segmentation, image reconstruction, and diagnoses [10–12]. In the musculoskeletal field, CNN has been used to diagnose fractures [13, 14], anterior cruciate ligament tears [15, 16], and developmental dysplasia of the hip [17]. Notably, CNN models in these studies have achieved equivalent or better diagnostic performance compared with doctors, indicating the feasibility of CNNs in orthopedics. However, previous CNN applications for RCTs have several limitations. At first, there were relatively few articles on the automatic diagnosis of supraspinatus tears [18–20], and clinical evaluation and correlation to surgical findings still need to be performed. Recent studies have also used machine learning to predict the surgical outcome of rotator cuff repair [21]. This hinders the application of artificial intelligence in the field of rotator cuff diseases, and the true capabilities of CNN for diagnosing rotator cuff tears in a clinical setting still need to be determined. Secondly, some studies have focused on model training based on X-ray or ultrasound screening, which cannot directly reflect rotator cuff tears [22–24]. In order to fill all these gaps, clinically validate the deep learning-based model performance, and improve diagnostic efficiency, we developed a CNN model for automated ST diagnosis based on MRI data. The diagnostic performance was evaluated by comparing four radiologists and four orthopedic surgeons with varied experiences on internal and surgery test datasets. Finally, the robustness of our proposed CNN model was validated by subgroup analyses for MRI data with different magnetic field strengths (MFSs) and degrees of tears.

## Materials and methods

### Data collection and MRI acquisition

**Fig. 1:**
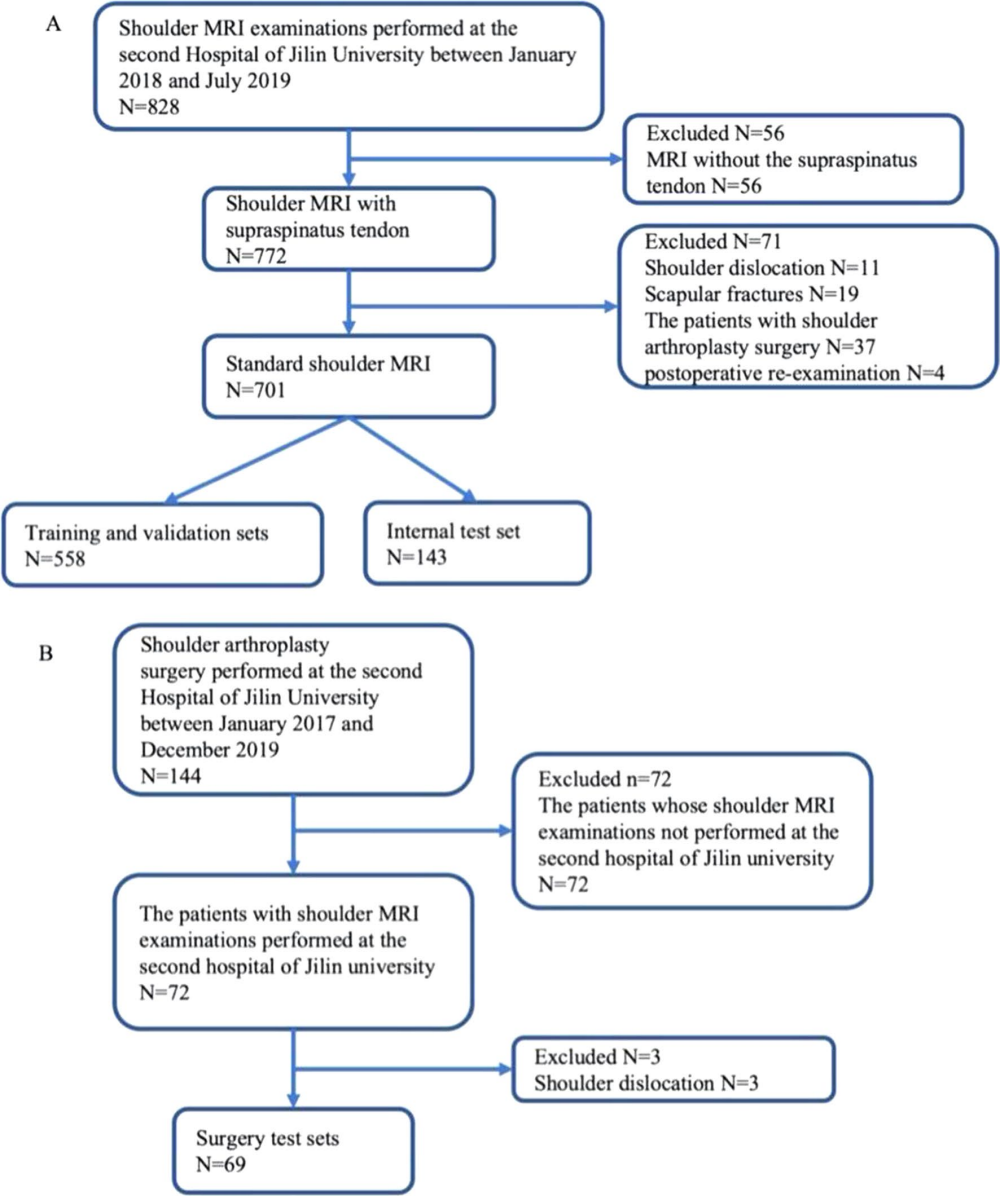
Experimental design. **A** Flowchart of the criteria applied to the enrolled patients with susceptible STs who underwent MRI. **B** Flowchart of the criteria applied to the enrolled patients with susceptible STs who underwent MRI examinations and shoulder arthroplasty surgery. ST, supraspinatus tear

This retrospective study was approved by the institutional review board of the Second Hospital of Jilin University (No. 2018247). The requirement for informed consent was waived since the patients’ information was anonymized to ensure privacy. Our study established three datasets: the training and validation set, internal testing set, and surgical test set. The establishment of the training and validation set and internal testing set was done simultaneously with the formation of the primary cohort. For the primary cohort, shoulder MRI data were collected from patients who received treatment in our hospital between January 2018 and October 2019 (*n* = 829). Ultimately, 701 shoulder scans were enrolled according to the inclusion and exclusion criteria (Fig. 1A) and further randomly divided into training and validation sets (558 shoulders) and an internal test set (143 shoulders). The training/validation set is primarily used for model training, while the internal testing set is used to evaluate and compare the diagnostic performance of the models and to make initial comparisons with the diagnoses made by human experts. The surgery test set is primarily based on another clinical cohort. For this particular cohort, shoulder MRI data with an arthroscopy diagnosis result were collected from patients who underwent shoulder arthroplasty surgery in our hospital between January 2017 and December 2019 (*n* = 144) and constituted another surgery test set (69 shoulders) after applying the exclusion criteria (Fig. 1B). The purpose of establishing the surgery test set is to utilize arthroscopy diagnosis as the definitive benchmark for objectively comparing diagnostic performance between the model and human experts.

In this study, digital radiography of the shoulder was taken using different system, including a 3.0 T GE MRI system and a 1.5 T Philips MRI systems. Oblique coronary fat sat proton density series (3.0 T) or oblique coronary proton density-weighted spectral attenuated inversion recovery series (1.5 T) was extracted from each examination for labeling and modeling [25].

### Reference standard establishment

Two experts Musculoskeletal (MSK) radiologists with more than 15 years of experience reviewing all 701 MRI scans, and their consensus was regarded as the reference standard (gold standard); a third senior expert MSK radiologist with 21 years’ experience made final judgments when there was a disagreement.

According to the reviewing criteria [26, 27], the MRI scans were annotated as usual (no tears) or tears, and the tears were further classified into partial-thickness tears and full-thickness tears. Partial-thickness tears include bursal-sided, articular, and interstitial tears; full-thickness tears include small, medium, giant, and massive tears based on severity. Concerning MRI data in the surgery test set, a senior orthopedist with more than 15 years of experience established the reference standard based on arthroscopy videos and patient surgical records.

After annotating the reference standard (usual or tears) by expert MSK radiologists or senior orthopedists, the region of interest (ROI) was manually segmented by a junior orthopedist with five years’ experience on the shoulder coronal image with the aid of labeling tools integrated with the InferScholar Center (Infervision, Beijing, China). The supraspinatus tendon and the insertion of the supraspinatus tendon on the humeral head were contained in the annotated ROIs.

### CNN models

**Fig. 2:**
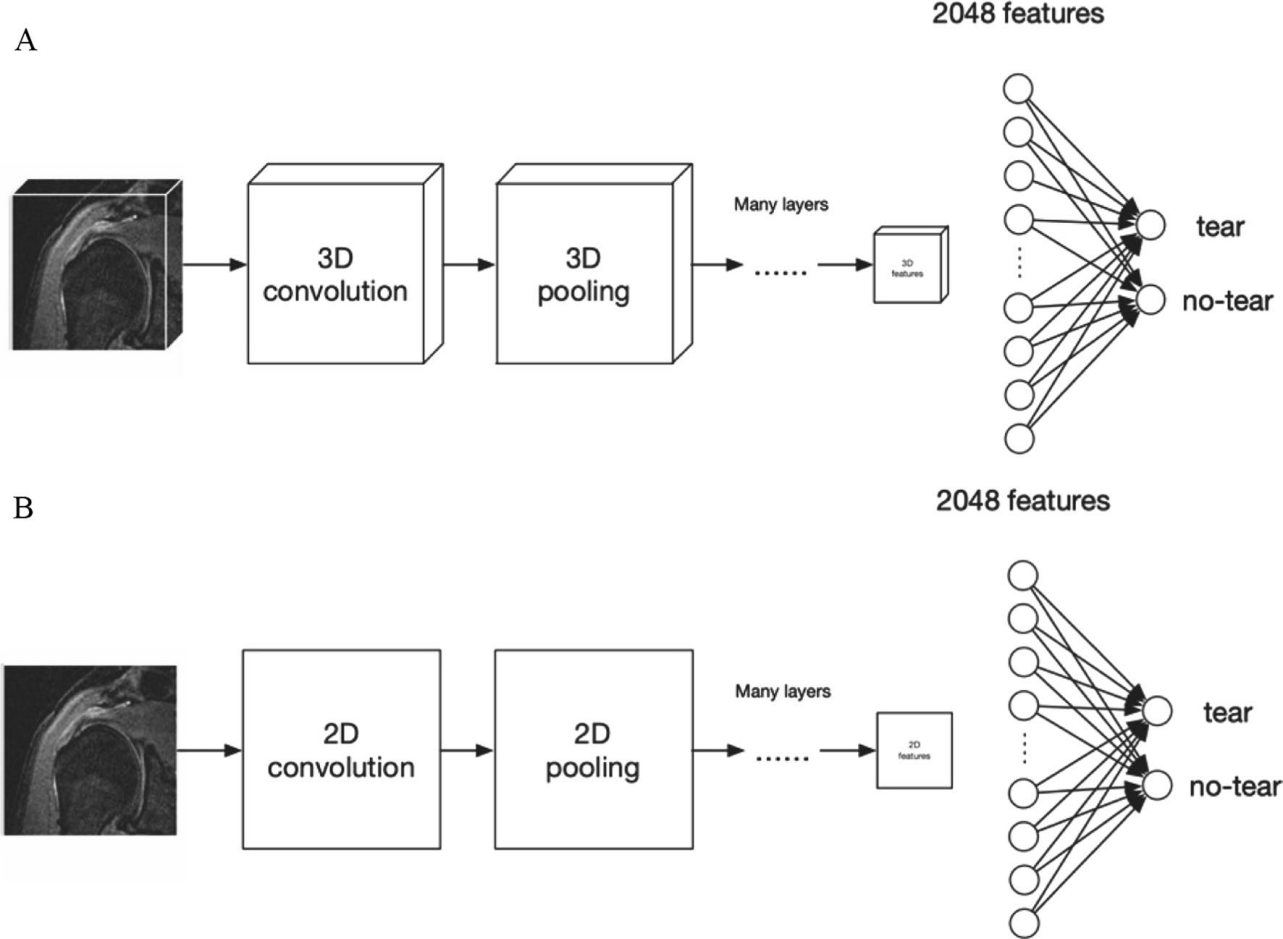
Schematic workflow of the 2D and 3D CNN models based on Xception. **A** For 2D CNN, a single shoulder slide was the input, while 2D convolution layers were utilized to extract image features. Finally, 2048 features were extracted and fed into a classifier, from which the output was the probabilities of tear and normal. **B** For the 3D CNN model, 3D shoulder image blocks were the input, and 3D convolution layers were utilized to extract image features. Finally, 2048 features were extracted and fed into a classifier, from which the output was the probabilities of tear and normal. CNN, convolutional neural network

This study established 2D and 3D model architectures based on Xception [28], a net utilizing depthwise separable convolution to perform feature extraction, which is more efficient. A total of 2048 dimensional features were extracted based on these architectures. The model classifier then took the extracted image features as input, and two nodes in Fig. 2 represent predicted output indicating tear or normal (no tear). The schematic workflow of the 2D and 3D models utilized is shown in Fig. 2. The main differences between 2 and 3D CNN are as follows. The 2D CNN employed 2D convolution and pooling layers fed with 2D pixel matrixes each time. With regard to the supraspinatus tendon on MRI, the sample unit was set as a rectangular region in a slice. Meanwhile, 3D CNN employed 3D convolution and pooling layers, and the sample unit was a cuboid block consisting of several MRI slices. Diagnostic analysis of 2D networks is based on a single slice, while 3D networks can obtain more information from a slice context and are more similar to the diagnostic process of radiologists considering the whole picture. For image classification tasks, a multilayer perception was connected to the features vector and served as a classifier.

### Data transformation

Limited by CNN convolution kernels and full-connected layer architectures, all input images must be resized to a fixed one. We used linear interpolation to resize all the images. The input size was 224 × 224 for 2D models and 128 × 128 × 64 for 3D models. In order to solve the problem of small data volume, augmentation techniques were applied. Brightness, contrast, and saturation were shifted within 30% to augment the gray value distribution. Random image flipping was also performed, which could ensure the model focuses more on the lesion's shape. No window width and level were set for MRI images. Pixel values were normalized at a mean of 0.456 and a standard error of 0.224 to accelerate the gradient descent process in training.

### Modeling

During the training process, Adam optimizer was used to achieve the best performance of the models, in which the beta1 and beta2 were set as 0.9 and 0.99, respectively. Cross-Entropy was selected to define the loss function in this study. Because of the imbalanced sizes between tear and normal (no tear) samples, 3.5 times weight was added to the losses of positive samples (tear). The weight decay was set as 0.000005 to avoid over-fitting to some extent. Furthermore, training data were shuffled to mitigate correlations of consecutive samples. At the beginning of training, Xavier was used to initialize model weights. The learning rate was set to 0.0001 and 0.00005 for 2D and 3D models, respectively. Decay of rate was 0.6 every 50 epochs. The training process ended when losses did not decrease any longer. Training curves are shown in Additional file 1: Fig. S1.

### Evaluation of diagnostic performance

The performances of these trained CNN-based models were evaluated and compared in terms of sensitivity, specificity, precision, accuracy, F1-score, Youden Index, and receiver operating characteristic (ROC) curves: F1-score = 2 × precision × sensitivity/(precision + sensitivity); Youden Index = sensitivity + specificity − 1.

To evaluate the feasibility and efficiency of the proposed CNN models in clinical practice, their diagnostic performances were compared with that of 4 radiologists and 4 orthopedic surgeons with varied experiences working at the Second Hospital of Jilin University who assessed the internal and surgery test sets. The total time taken by the clinical expert to interpret the MRI were recorded.

### Statistical analyses

SPSS 21.0 (IBM, Armonk, NY, USA) was utilized for statistical analyses. The diagnostic performances of CNN-based models in the discrimination of normal tendons and STs were evaluated by ROC curve analyses. The power analysis method was chosen as post hoc analysis. The threshold that resulted in the highest G-Mean score in the validation set was selected as the optimal diagnostic threshold; the sensitivity, specificity, precision, accuracy, and Youden-Index were then calculated based on it. Statistical analyses were performed by Pearson’s c2 tests between different models and test datasets. The exact Fleiss kappa is reported to assess the level of agreement of the eight clinical experts. Kappa analysis was used to assess the diagnostic performance between clinicians. For all tests, *p* < 0.05 was considered statistically significant.

## Results

### Patient characteristics in grouped datasets

Of the retrospectively enrolled 770 MRI scans, 701 were divided into a training and validation set (*n* = 558) and internal test set (*n* = 143), while the remaining 69 MRI scans from patients who underwent shoulder arthroplasty surgery were considered a separate test dataset. The clinical characteristics of enrolled patients in each dataset, including age, sex, tear sides, MFS, and tear classification, are listed in Table 1. Briefly, among the 770 patients, 483 (62.7%) underwent examinations using 1.5 T MRI systems, and 230 patients (29.9%) were diagnosed with STs. Among these 230 patients, 110 (47.8%) and 120 (52.2%) were diagnosed with partial- and full-thickness tears, respectively. Representative images of different ST subtypes are shown in Additional file 2: Fig. S2.

**Table 1:** Clinical characteristics of 770 shoulders in grouped datasets

Characteristic	Training and validation sets	Internal test sets	Surgery test sets
No. of shoulders	558	143	69
Tear	157 (28.1)	50 (35.0)	23 (33.3)
No tear	401 (71.9)	93 (65.0)	46 (66.7)
Sex			
F	244 (43.7)	80 (55.9)	37 (53.6)
M	314 (56.3)	63 (44.1)	32 (46.4)
Age*	50.1 (10, 85)	49.4 (9, 81)	54.7 (17, 78)
0–39	108 (19.4)	34 (23.8)	11 (15.9)
40–49	126 (22.6)	27 (18.9)	9 (13.0)
50–59	196 (35.1)	44 (30.8)	20 (29.0)
60–89	128 (22.9)	38 (26.6)	29 (42.0)
Right	296 (53.0)	70 (49.0)	36 (52.2)
Left	262 (47.0)	73 (51.0)	33 (47.8)
Magnetic field strength			
1.5 T	365 (65.4)	96 (67.1)	22 (31.9)
3 T	193 (34.6)	47 (32.9)	47 (68.1)
Classification of tears			
Partial-thickness tears	81 (14.5)	25 (17.5)	3 (4.3)
Full-thickness tears	76 (13.6)	25 (17.5)	20 (29.0)
Small tears	47 (8.4)	12 (8.4)	8 (11.6)
Medium tears	20 (3.6)	7 (4.9)	6 (8.7)
Large tears	5 (0.9)	5 (3.5)	6 (8.7)
Massive tears	4 (0.7)	1 (0.7)	0 (0)

Unless otherwise specified, data in parentheses are percentages

*Numbers in parentheses are the ranges

### Diagnostic performance of the 2D and 3D CNN models

**Fig. 3:**
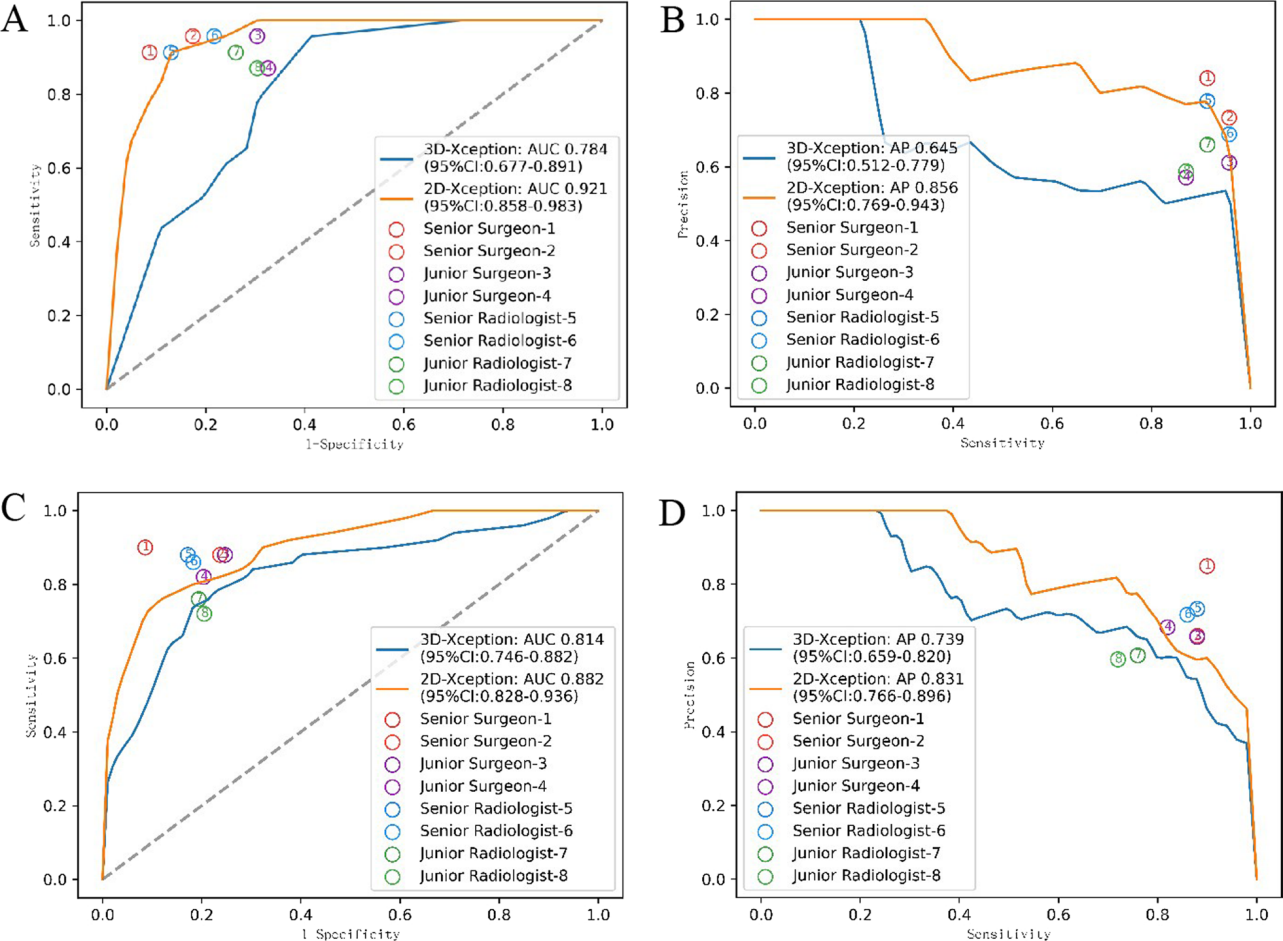
Diagnostic performance of the models and the reading clinicians. **A** The diagnostic performance of 8 clinicians was dotted in the ROC curves of the 2D and 3D CNN models according to the sensitivity and specificity for the surgery test set. **B** The diagnostic performance of 8 clinicians was dotted in the Precision and Recall (PR) curves of the 2D and 3D CNN models according to the precision and sensitivity for the surgery test set. **C** The diagnostic performance of 8 clinicians was dotted in the ROC curves of the 2D and 3D CNN models according to the sensitivity and specificity for the internal test set. **D** The diagnostic performance of 8 clinicians was dotted in the PR curves of the 2D and 3D CNN models according to the precision and sensitivity for the surgery test set on the internal test set

To ensure adequate diagnostic performance, we trained two models at the slice level (2D model) and volume level (3D model) and evaluated them on surgery and internal test datasets. Concerning the area under the ROC curve (AUC) of 2D and 3D models, 0.882 (95% confidence interval [CI] [0.817, 0.947]) and 0.814 (95% CI [0.735, 0.893]) were achieved on the internal test set (Fig. 3C) while 0.921 (95% CI [0.841, 1.000]) and 0.784 (95% CI [0.661, 0.907]) were obtained on surgery test set (Fig. 3A), respectively. More detailed diagnostic metrics are listed in Table 2 and Additional file 3: Table S1, and 2-class confusion matrices of models on the test sets are provided in Additional file 4: Fig. S3. The 2D CNN model generally outperformed the 3D model on both test sets. The 2D CNN model achieved a sensitivity of 0.78, specificity of 0.84, and F1-score of 0.75 on the internal test set and a sensitivity of 0.91, specificity of 0.85, and F1-score of 0.82 on the surgery test set. An overall better diagnostic performance was found in the surgery test set. Notably, it took these CNN models just 0.17 s to make a diagnosis, which was considered efficient. According to previous studies, we assumed that the specificity of young surgeons in diagnosing rotator cuff tears was 0.636 [29]. Based on our results, we hypothesized that the specificity of the 2D model would be higher than that of young doctors on the surgery test set, with a specificity of 0.870 for the 2D model. With the given effect size and alpha error of 0.05, a power of 0.98 was calculated.

**Table 2:** Diagnostic performance of 2D and 3D CNN models and participated reading clinicians on surgery and internal test sets

Metrics	Accuracy	Precision	Sensitivity	Specificity	F1-score	Youden-Index
Surgery test set						
2D CNN	0.87 (60/69)	0.75 (21/28)	0.913 (21/23)	0.848 (39/46)	0.824	0.761
3D CNN	0.71 (49/69)	0.54 (17/31)	0.739 (17/23)	0.696 (32/46)	0.624	0.435
Senior surgeon 1 and 2	0.891 (123/138)	0.782 (43/55)	0.935 (43/46)	0.870 (80/92)	0.852	0.805
Junior surgeon 3 and 4	0.761 (105/138)	0.592 (42/71)	0.913 (42/46)	0.685 (63/92)	0.718	0.598
Senior radiologist 5 and 6	0.862 (119/138)	0.729 (43/59)	0.935 (43/46)	0.826 (76/92)	0.819	0.761
Junior radiologist 7 and 8	0.775 (107/138)	0.612 (41/67)	0.891 (41/46)	0.717 (66/92)	0.726	0.608
Internal test set						
2D CNN	0.818 (117/143)	0.72 (39/54)	0.78 (39/50)	0.839 (78/93)	0.75	0.619
3D CNN	0.783 (112/143)	0.679 (36/53)	0.72 (36/50)	0.817 (76/93)	0.699	0.537
Senior surgeon 1 and 2	0.857 (245/286)	0.748 (89/119)	0.89 (89/100)	0.839 (156/186)	0.813	0.729
Junior surgeon 3 and 4	0.801 (229/286)	0.669 (85/127)	0.85 (85/100)	0.753 (140/186)	0.749	0.603
Senior radiologist 5 and 6	0.839 (240/286)	0.725 (87/120)	0.87 (87/100)	0.823 (153/186)	0.791	0.693
Junior radiologist 7 and 8	0.759 (217/286)	0.637 (72/113)	0.74 (74/100)	0.801 (149/186)	0.685	0.541

**Fig. 4:**
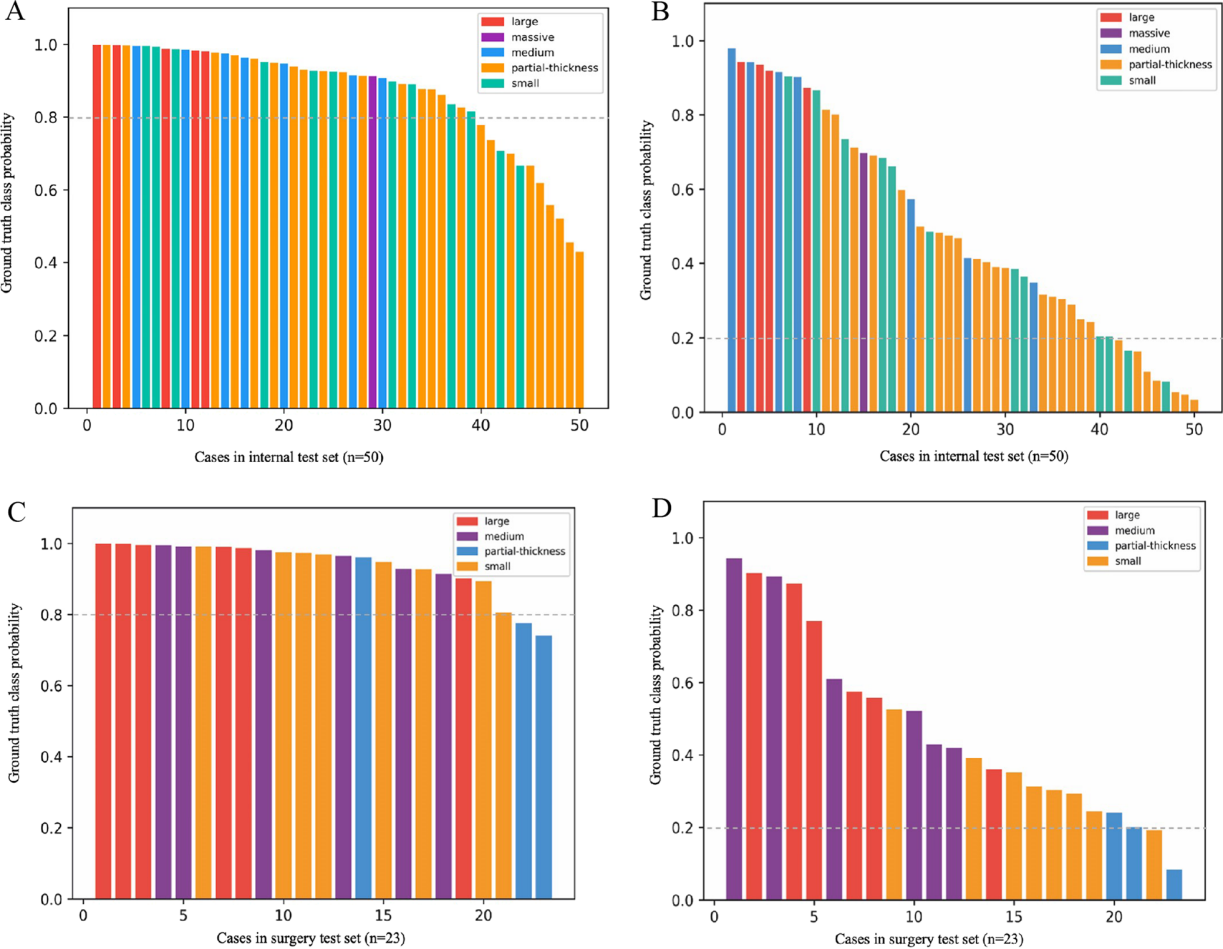
Predicted probabilities for different ST subtypes by the 2D CNN model. To determine the error-prone ST subtypes for 2D and 3D CNN models, the predicted probabilities for each patient in internal (**A** and **B**) and surgery (**C** and **D**) test sets were calculated and ranked accordingly. The dotted lines represent the threshold of the corresponding model (0.8 for 2D and 0.2 for 3D). ST subtypes are presented in different colors

Although the binary classification models were trained in this study, we also assessed the diagnostic performance for ST subtypes to determine the error-prone subtypes by analyzing the predicted probabilities of each case. Specifically, the partial-thickness tears were easily missed by 2D CNN models, while partial-thickness tears and small tears were the easily missed subtypes for the 3D CNN model (Fig. 4). The detection rates of partial-thickness and small tears by the 2D CNN model reached 0.625 and 0.833 on the internal test set compared to 0.33 and 1.00 on the surgery test set, indicating a potential role of the 2D CNN model in diagnosing STs at early stages.

**Fig. 5:**
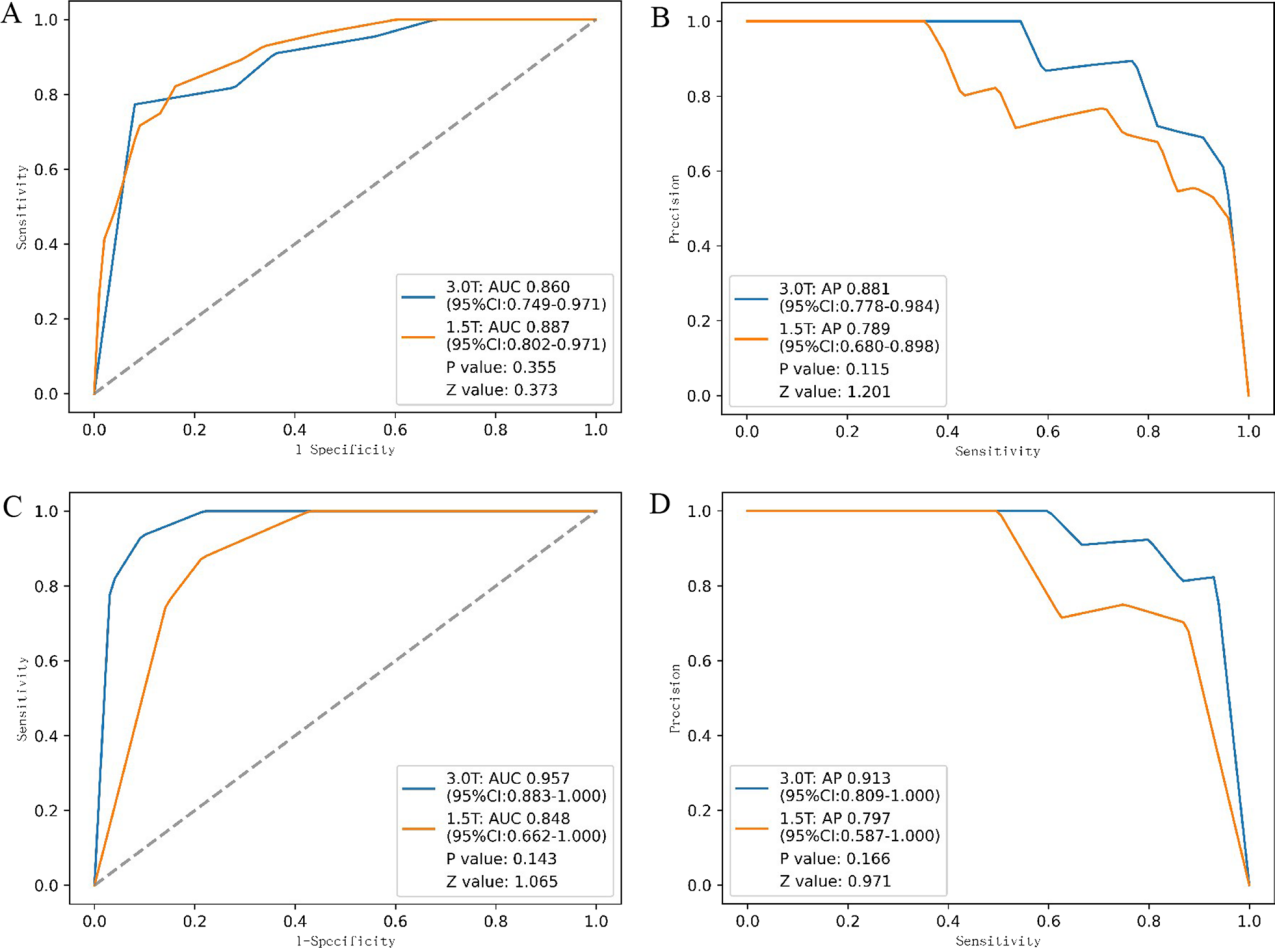
ROC and PR curves of the 2D CNN model on 1.5 T and 3.0 T MRI examinations. **A** ROC curves of the 2D CNN model for 1.5 T and 3.0 T MRI examinations on internal test set; **B** PR curves of the 2D CNN model for 1.5 T and 3.0 T MRI examinations on the internal test set; **C** ROC curves of the 2D CNN model for 1.5 T and 3.0 T MRI examinations on the surgery test set; **D** PR curves of the 2D CNN model for 1.5 T and 3.0 T MRI examinations on the surgery test set. CNN, convolutional neural network; ST, supraspinatus tear; ROC, receiver operating characteristic

Another subgroup analysis was performed to evaluate the robustness of these models for MRI systems with different MFSs (1.5 T and 3.0 T). As shown in Fig. 5 and Additional file 5: Table S2, the 2D and 3D CNN models showed no significant difference in diagnostic performance between 1.5 and 3 T MRI data on either the internal or surgery test sets.

### Diagnostic performance of clinical experts

A total of 8 clinical experts, including 4 radiologists and 4 orthopedic surgeons with varied experiences, participated in the reader study. The inter-rater agreement among the 8 clinical experts, measured by the exact Fleiss kappa score, was 0.575 on the surgery test sets and 0.586 on the internal test sets. The diagnostic metrics details are listed in Table 2, Additional file 3: Table S1 and Additional file 5: Table S2. The 2D CNN model generally exhibited an equivalent diagnostic performance to the senior clinicians and a better one than junior clinicians (Fig. 3). In addition, it took the clinicians a mean of 39 s to make a diagnosis.

## Discussion

In this study, a 2D CNN model was developed to discriminate normal tendons and STs on shoulder MRI and achieved an AUC of 0.921. In addition to the reduced assessment time, the diagnostic performance of 2D CNN model was equivalent to that of senior clinicians, supporting the clinical assistance potentials of the 2D CNN model.

Internal and surgery test sets were utilized to evaluate the models’ diagnostic performance. In addition, 2D CNN model achieved a better performance than radiologists and orthopedic surgeons on the surgery set, which validated model’s clinically usability. On one hand, there were more obvious ST manifestations in the surgery set. On the other hand, in contrast to the solid evidence in the surgery videos, the reference standard for MRI data in the training and validation set was annotated by the consensus of MSK radiologists, while the surgery videos could decouple the labels from reader opinions and result in different diagnostic performance of the 2D model between internal and surgery test sets.

The best performance was achieved by the 2D CNN model on either internal or surgery test set, which might have been related to the signal-to-noise (S/N) ratio of tears in different models. Notably, each slide was labeled as “normal” or “tear” for the 2D model, whereas the volume consisted of different slides and was labeled only once for the 3D model with the same ROI, normal tendon, or tear. The S/N ratio of the ROI for the slice-wise 2D model was much greater compared with the whole volume; a single image was inputted into the 2D model sequentially, and a positive diagnosis was made as long as one of the single slides containing tears was recognized. In contrast, the labeled volume was the input for the 3D model and the lesions took up only a small portion of the whole imaging volume, so the lower S/N ratio would probably weaken the diagnostic performance of the 3D model.

Previous studies showed that the sensitivity and specificity of MRI for diagnosing rotator cuff tears ranged from 0.83 to 0.977 and 0.636 to 0.91, respectively [29–31], and these values were highly related to the experience or professionalism of the reading experts [9, 32]. In comparison, the proposed 2D model exhibited a sensitivity of 0.91 and a specificity of 0.85 on the surgery test set, indicating an equivalent diagnostic performance to the mainstream studies. This suggests that the 2D model could be an efficient option for community hospital radiologists to obtain a state-of-the-art reference. It is worth noting that, in general, although the model did not perform as well as the senior orthopedic surgeons, it is better than most junior doctors.

Previous studies showed that the improvement in diagnostic performance of clinicians benefit from targeted training rather than experience growth [33, 34]. In primary medical institutions, clinicians rarely have the opportunity to receive systematic and targeted MRI interpretation training. Therefore, the 2D CNN model showed considerable potentials to assist inexperienced clinicians to diagnose STs in clinical practice, as evidenced by a better diagnostic performance than junior clinicians.

In addition to discrimination between normal tendons and STs, we also analyzed the diagnostic performance for different subtypes of tears which were classified based on the severity. A recent meta-analysis reported that the pooled sensitivities for partial- and full-thickness tear diagnoses on MRI were 0.70 (95% CI 0.50–0.85) and 0.81 (95% CI 0.69–0.89), respectively [35]. Although the number of cases in our study was much smaller, the 2D CNN model exhibited the sensitivity up to 0.625 and 0.833 in detecting partial-thickness and small tears, respectively, indicating the potentials in detecting early stage STs. To illustrate the robustness of the 2D model, we further explored its diagnostic performance on MRI with different MFSs in the test sets. No statistical difference in diagnostic performance was found between 1.5 and 3.0 T MRI data, indicating its potential to be widely adapted in clinical practice.

Some studies have already applied deep learning to the processing of shoulder rotator cuff injury imaging. Previous studies which applied CNN to supraspinatus tendon injuries focused on either assisting doctors to evaluate the recovery of patients after surgery by automatic segmentation of supraspinatus and calculation of supraspinatus volume on MRI [24] or evaluating postoperative prognosis by automatically determining ST severity, the occupation ratio stage, and the Goutallier grade [23]. Another recent study employed CNN to rule out severe RCTs in the X-ray images-based screening and achieved a negative predictive value of 0.966 [22]. In contrast, we directly proposed a 2D CNN model to diagnose STs on MRI which was validated as an adequate, efficient, and robust tool for clinicians. In addition, unlike the diagnostic application of rotator cuff injuries, the study conducted by Potty et al. utilized machine learning methods to incorporate patient demographics, comorbidities, rotator cuff tears, tissue quality, and other parameters. The prediction of shoulder joint function after arthroscopic rotator cuff repair has yielded satisfactory results, thereby broadening the application of artificial intelligence in the health management of rotator cuff injuries [21].

To our knowledge, three studies have automatically applied deep learning to classify supraspinatus muscle injuries. Similar to our study, these studies have achieved excellent diagnostic performance of the models, with AUC ranging from 0.910 to 0.93. In comparison with the study by Lin et al. [20], although they established a larger-scale dataset, they needed a more objective evaluation of model diagnostic performance based on a dataset established with arthroscopic surgery as the gold standard. Compared to the study by Jason et al. [18], apart from lacking objective validation with a standard gold dataset, their research lacked comparing diagnostic performance with experts, which would objectively evaluate the model's performance. In the previous study by Shim et al. [19], they also established a larger-scale dataset but only evaluated the difference in diagnostic performance between the model and orthopedic doctors. In contrast, our study compared the model's diagnostic performance with clinicians of different experience levels, including orthopedic doctors and radiologists. It concluded that the model's performance was superior to that of less experienced clinicians. Additionally, a similarity among these studies is that there was no significant difference in the diagnostic performance of the models between 3 and 1.5 T.

There are several limitations to this study. Firstly, this was a retrospective study at a single institution, which may limit the generalizability of the results. Secondary, there is possible bias of reference standard introduced from readers’ opinion and the relatively small training dataset, although the transfer learning technique was employed to ensure maximum training efficiency. There is no doubt that utilization of larger training datasets in future studies may further improve the diagnostic performance of the ST classification system. In addition, MRI data collected from other hospitals would also enable the generalizability of the selected 2D CNN model.

## Conclusions

In summary, the proposed advanced convolutional neural network (CNN) based on Xception, for differentiating STs from normal tendons based on MRI achieved an AUC of 0.924; the diagnostic performance was equivalent to senior clinicians and better than junior clinicians. Our results support that CNN algorithms can be successfully applied to advanced skeletal muscle images to generate rapid automated diagnoses and improve clinical workflow efficiency.

## Supplementary Information

**Additional file 1: Figure S1** Training curves of the 2D (A) and 3D (B) CNN models.

**Additional file 2: Figure S2** Different ST subtypes. (a) Histological damage diagram. (b) Representative images onMRI and arthroscopy. ST, supraspinatus tear.

**Additional file 3: Table S1** Diagnostic performance of 2D and 3D CNN models and participated reading clinicians on surgery andinternal test sets.

**Additional file 4: Figure S3** 2-class confusion matrices of models on test set. (A) 2-class confusionmatrices of 2D model on internal test set. (B) 2-class confusion matrices of 3D model on internaltest set. (C) 2-class confusion matrices of 2D model on surgery test set. (D) 2-class confusionmatrices of 3D model on surgery test set. CNN, convolutional neural network; ROC, receiveroperating characteristic.

**Additional file 5: Table S2** Diagnostic performance of 2D CNN models and reading clinicians on 1.5T and 3.0T MRIexaminations.

## Data Availability

The data presented in this study are available on request from the corresponding author. The data are not publicly available due to the restriction of IRB.

## Abbreviations

AUC: Area under the ROC curve
CNN: Convolutional neural network
MFS: Magnetic field strength
RCT: Rotator cuff tear
ROC: Receiver operating characteristic
ROI: Region of interest
S/N: Signal-to-noise
ST: Supraspinatus tear
MSK: Musculoskeletal

## References

[1] Morag Y, Jacobson JA, Miller B, De Maeseneer M, Girish G, Jamadar D (2006). MR imaging of rotator cuff injury: what the clinician needs to know. Radiographics.

[2] Minagawa H, Yamamoto N, Abe H, Fukuda M, Seki N, Kikuchi K, Kijima H, Itoi E (2013). Prevalence of symptomatic and asymptomatic rotator cuff tears in the general population: from mass-screening in one village. J Orthop.

[3] El-Azab H, Buchmann S, Beitzel K, Waldt S, Imhoff AB (2010). Clinical and structural evaluation of arthroscopic double-row suture-bridge rotator cuff repair: early results of a novel technique. Knee Surg Sports Traumatol Arthrosc.

[4] Clark JC, Ritchie J, Song FS, Kissenberth MJ, Tolan SJ, Hart ND, Hawkins RJ (2012). Complication rates, dislocation, pain, and postoperative range of motion after reverse shoulder arthroplasty in patients with and without repair of the subscapularis. J Shoulder Elbow Surg.

[5] Kijowski R, Thurlow P, Blankenbaker D, Liu F, McGuine T, Li G, Tuite M (2019). Preoperative MRI shoulder findings associated with clinical outcome 1 year after rotator cuff repair. Radiology.

[6] Moosmayer S, Lund G, Seljom US, Haldorsen B, Svege IC, Hennig T, Pripp AH, Smith HJ (2019). At a 10-year follow-up, tendon repair is superior to physiotherapy in the treatment of small and medium-sized rotator cuff tears. J Bone Joint Surg Am.

[7] Kwong CA, Ono Y, Carroll MJ, Fruson LW, More KD, Thornton GM, Lo IK (2019). Full-thickness rotator cuff tears: what is the rate of tear progression? A systematic review. Arthrosc J Arthrosc Relat Surg.

[8] Teefey SA, Rubin DA, Middleton WD, Hildebolt CF, Leibold RA, Yamaguchi K (2004). Detection and quantification of rotator cuff tears: comparison of ultrasonographic, magnetic resonance imaging, and arthroscopic findings in seventy-one consecutive cases. JBJS.

[9] Theodoropoulos JS, Andreisek G, Harvey EJ, Wolin P (2010). Magnetic resonance imaging and magnetic resonance arthrography of the shoulder: dependence on the level of training of the performing radiologist for diagnostic accuracy. Skeletal Radiol.

[10] Zhou L-Q, Wu X-L, Huang S-Y, Wu G-G, Ye H-R, Wei Q, Bao L-Y, Deng Y-B, Li X-R, Cui X-W (2020). Lymph node metastasis prediction from primary breast cancer US images using deep learning. Radiology.

[11] Lakhani P, Sundaram B (2017). Deep learning at chest radiography: automated classification of pulmonary tuberculosis by using convolutional neural networks. Radiology.

[12] Norman B, Pedoia V, Majumdar S (2018). Use of 2D U-Net convolutional neural networks for automated cartilage and meniscus segmentation of knee MR imaging data to determine relaxometry and morphometry. Radiology.

[13] Langerhuizen DWG, Janssen SJ, Mallee WH, van den Bekerom MPJ, Ring D, Kerkhoffs G, Jaarsma RL, Doornberg JN (2019). What Are the applications and limitations of artificial intelligence for fracture detection and classification in orthopaedic trauma imaging? A systematic review. Clin Orthop Relat Res.

[14] Derkatch S, Kirby C, Kimelman D, Jozani MJ, Davidson JM, Leslie WD (2019). Identification of vertebral fractures by convolutional neural networks to predict nonvertebral and hip fractures: a registry-based cohort study of dual x-ray absorptiometry. Radiology.

[15] Liu F, Guan B, Zhou Z, Samsonov A, Rosas H, Lian K, Sharma R, Kanarek A, Kim J, Guermazi A (2019). Fully automated diagnosis of anterior cruciate ligament tears on knee MR images by using deep learning. Radiol Artif Intell.

[16] Bien N, Rajpurkar P, Ball RL, Irvin J, Park A, Jones E, Bereket M, Patel BN, Yeom KW, Shpanskaya K (2018). Deep-learning-assisted diagnosis for knee magnetic resonance imaging: development and retrospective validation of MRNet. PLoS Med.

[17] Li Q, Zhong L, Huang H, Liu H, Qin Y, Wang Y, Zhou Z, Liu H, Yang W, Qin M (2019). Auxiliary diagnosis of developmental dysplasia of the hip by automated detection of Sharp's angle on standardized anteroposterior pelvic radiographs. Medicine (Baltimore).

[18] Yao J, Chepelev L, Nisha Y, Sathiadoss P, Rybicki FJ, Sheikh AM (2022). Evaluation of a deep learning method for the automated detection of supraspinatus tears on MRI. Skeletal Radiol.

[19] Shim E, Kim JY, Yoon JP, Ki SY, Lho T, Kim Y, Chung SW (2020). Automated rotator cuff tear classification using 3D convolutional neural network. Sci Rep.

[20] Lin DJ, Schwier M, Geiger B, Raithel E, von Busch H, Fritz J, Kline M, Brooks M, Dunham K, Shukla M (2023). Deep learning diagnosis and classification of rotator cuff tears on shoulder MRI. Invest Radiol.

[21] Potty AG, Potty ASR, Maffulli N, Blumenschein LA, Ganta D, Mistovich RJ, Fuentes M, Denard PJ, Sethi PM, Shah AA (2023). Approaching artificial intelligence in orthopaedics: predictive analytics and machine learning to prognosticate arthroscopic rotator cuff surgical outcomes. J Clin Med.

[22] Kim Y, Choi D, Lee KJ, Kang Y, Ahn JM, Lee E, Lee JW, Kang HS (2020). Ruling out rotator cuff tear in shoulder radiograph series using deep learning: redefining the role of conventional radiograph. Eur Radiol.

[23] Kim JY, Ro K, You S, Nam BR, Yook S, Park HS, Yoo JC, Park E, Cho K, Cho BH (2019). Development of an automatic muscle atrophy measuring algorithm to calculate the ratio of supraspinatus in supraspinous fossa using deep learning. Comput Methods Programs Biomed.

[24] Kim S, Lee D, Park S, Oh K-S, Chung SW, Kim Y (2017). Automatic segmentation of supraspinatus from MRI by internal shape fitting and autocorrection. Comput Methods Programs Biomed.

[25] Singson R, Hoang T, Dan S, Friedman M (1996). MR evaluation of rotator cuff pathology using T2-weighted fast spin-echo technique with and without fat suppression. AJR Am J Roentgenol.

[26] Cofield RH, Parvizi J, Hoffmeyer PJ, Lanzer WL, Ilstrup DM, Rowland CM (2001). Surgical repair of chronic rotator cuff tears: a prospective long-term study. JBJS.

[27] Yoo J-S, Heo K, Park S-G, Ham H-J, Seo J-B (2019). The supraspinatus occupation ratios of both the ≥ 50% articular- and bursal-side partial-thickness rotator cuff tears were low and the infraspinatus occupation ratio of the ≥ 50% bursal-side partial-thickness rotator cuff tears was low. Knee Surg Sports Traumatol Arthrosc.

[28] Chollet F: Xception: deep learning with depthwise separable convolutions. In: Proceedings of the IEEE conference on computer vision and pattern recognition, vol. 2017. 2017; p. 1251–1258.

[29] Moosmayer S, Tariq R, Stiris MG, Smith H-J (2010). MRI of symptomatic and asymptomatic full-thickness rotator cuff tears: a comparison of findings in 100 subjects. Acta Orthop.

[30] Smith TO, Daniell H, Geere J-A, Toms AP, Hing CB (2012). The diagnostic accuracy of MRI for the detection of partial-and full-thickness rotator cuff tears in adults. Magn Reson Imaging.

[31] Blanchard T, Bearcroft P, Constant C, Griffin D, Dixon A (1999). Diagnostic and therapeutic impact of MRI and arthrography in the investigation of full-thickness rotator cuff tears. Eur Radiol.

[32] Wnorowski DC, Levinsohn EM, Chamberlain BC, McAndrew DL (1997). Magnetic resonance imaging assessment of the rotator cuff: is it really accurate?. Arthrosc J Arthrosc Relat Surg.

[33] Fischer MA, Mazor KM, Baril J, Alper E, DeMarco D, Pugnaire M (2006). Learning from mistakes. Factors that influence how students and residents learn from medical errors. J Gen Intern Med.

[34] Mankad K, Hoey ET, Jones JB, Tirukonda P, Smith JT (2009). Radiology errors: Are we learning from our mistakes?. Clin Radiol.

[35] Liu F, Cheng X, Dong J, Zhou D, Han S, Yang Y (2020). Comparison of MRI and MRA for the diagnosis of rotator cuff tears: a meta-analysis. Medicine.

