# Supplementary figures and images for: Development and clinical validation of deep learning for auto-diagnosis of supraspinatus tears
Source: J Orthop Surg Res. 2023 Jun 13;18:426. doi: 10.1186/s13018-023-03909-z (PMC10262398; doi:10.1186/s13018-023-03909-z)

Additional Files 1

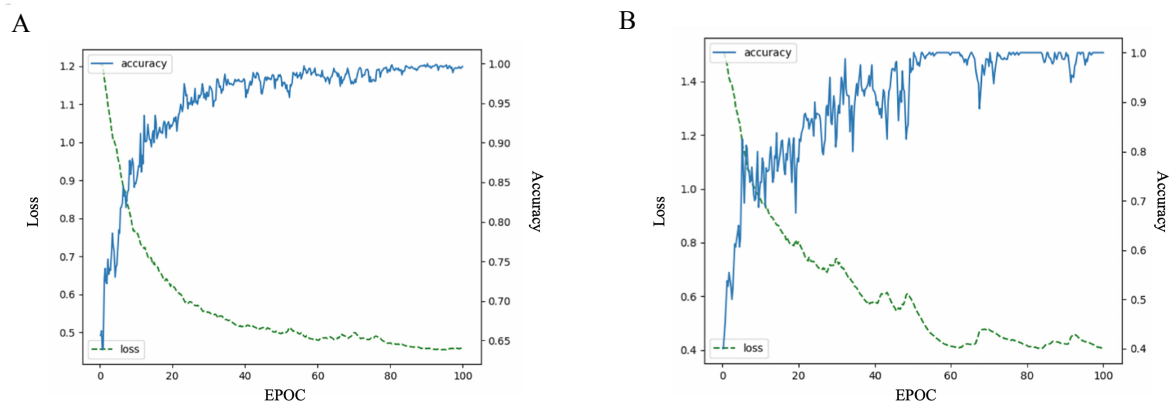

Additional figure 1. Training curves of the 2D (A) and 3D (B) CNN models.

Supplement: Supplementary file 1 — Additional file 1: Figure S1 Training curves of the 2D (A) and 3D (B) CNN models. [file 13018_2023_3909_MOESM1_ESM.pdf]
