# Supplementary material for: Development and clinical validation of deep learning for auto-diagnosis of supraspinatus tears
Source: J Orthop Surg Res. 2023 Jun 13;18:426. doi: 10.1186/s13018-023-03909-z (PMC10262398; doi:10.1186/s13018-023-03909-z)
Supplement: Supplementary file 2 — Additional file 2: Figure S2 Different ST subtypes. (a) Histological damage diagram. (b) Representative images onMRI and arthroscopy. ST, supraspinatus tear. [file 13018_2023_3909_MOESM2_ESM.pdf]

Additional Files 2

A

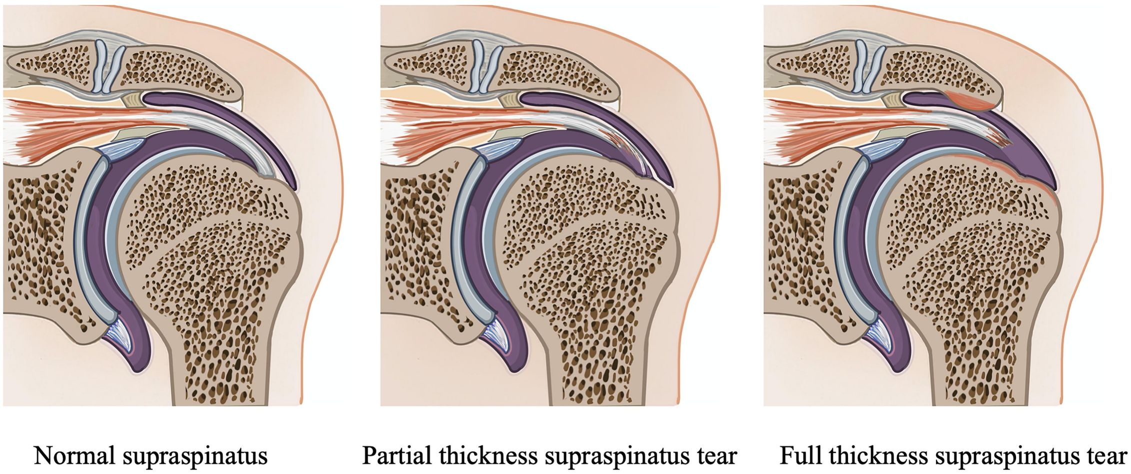

B

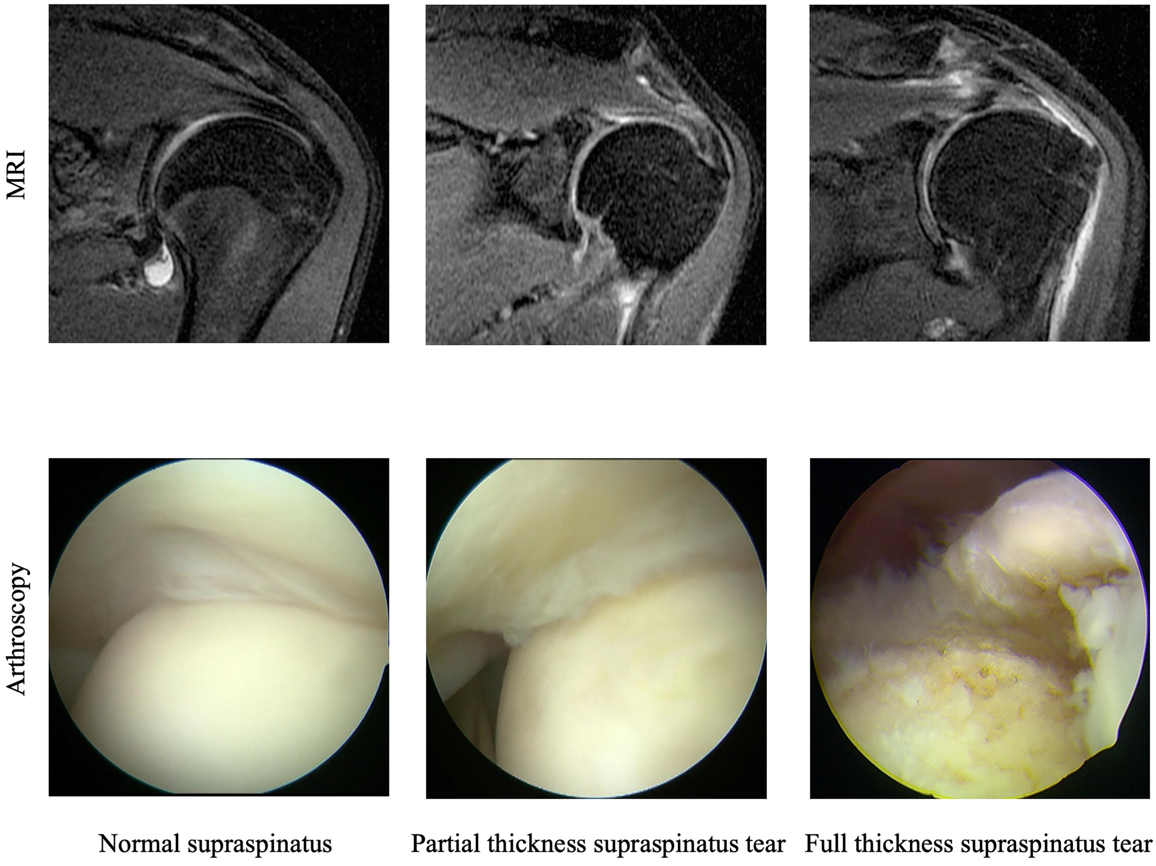

Additional Figure 2. Different ST subtypes. (a) Histological damage diagram. (b) Representative images on MRI and arthroscopy. ST, supraspinatus tear.
