# Supplementary material for: Development and clinical validation of deep learning for auto-diagnosis of supraspinatus tears
Source: J Orthop Surg Res. 2023 Jun 13;18:426. doi: 10.1186/s13018-023-03909-z (PMC10262398; doi:10.1186/s13018-023-03909-z)
Supplement: Supplementary file 3 — Additional file 3: Table S1 Diagnostic performance of 2D and 3D CNN models and participated reading clinicians on surgery andinternal test sets. [file 13018_2023_3909_MOESM3_ESM.pdf]

Additional Files 3

Additional Table 1. Diagnostic performance of 2D and 3D CNN models and participated reading clinicians on surgery and internal test sets.

	Metrics	Accuracy	Precision	Sensitivity	Specificity	F1-score	Youden-Index
Surgery test set	2D CNN	0.87 (60/69)	0.75 (21/28)	0.913 (21/23)	0.848 (39/46)	0.824	0.761
	3D CNN	0.71 (49/69)	0.54 (17/31)	0.739 (17/23)	0.696 (32/46)	0.624	0.435
	Senior Surgeon 1	0.913 (63/69)	0.84 (21/25)	0.913 (21/23)	0.913 (42/46)	0.875	0.826
	Senior Surgeon 2	0.869 (60/69)	0.733 (22/30)	0.957 (22/23)	0.826 (38/46)	0.83	0.783
	Junior Surgeon 3	0.782 (54/69)	0.611 (22/36)	0.957 (22/23)	0.696 (32/46)	0.746	0.653
	Junior Surgeon 4	0.739 (51/69)	0.571 (20/35)	0.870 (20/23)	0.674 (31/46)	0.689	0.544
	Senior Radiologist 5	0.884 (61/69)	0.778 (21/27)	0.913 (21/23)	0.870 (40/46)	0.84	0.783
	Senior Radiologist 6	0.841 (58/69)	0.688 (22/32)	0.957 (22/23)	0.783 (36/46)	0.801	0.74
	Junior Radiologist 7	0.797 (55/69)	0.636 (21/33)	0.913 (21/23)	0.739 (34/46)	0.75	0.652
	Junior Radiologist 8	0.753 (52/69)	0.588 (20/34)	0.870 (20/23)	0.696 (32/46)	0.702	0.566
Internal test set	2D CNN	0.818 (117/143)	0.72 (39/54)	0.78 (39/50)	0.839 (78/93)	0.75	0.619
	3D CNN	0.783 (112/143)	0.679 (36/53)	0.72 (36/50)	0.817 (76/93)	0.699	0.537
	Senior Surg eon 1	0.909 (130/143)	0.849 (45/53)	0.9 (45/50)	0.914 (85/93)	0.874	0.814
	Senior Surgeon 2	0.804 (115/143)	0.66 (44/66)	0.88 (44/50)	0.763 (71/93)	0.754	0.643
	Junior Surgeon 3	0.797 (114/143)	0.657 (44/67)	0.88 (44/50)	0.753 (70/93)	0.752	0.633
	Junior Surgeon 4	0.804 (115/143)	0.683 (41/60)	0.82 (41/50)	0.796 (74/93)	0.745	0.616
	Senior Radiologist 5	0.846 (121/143)	0.733 (44/60)	0.88 (44/50)	0.828 (77/93)	0.8	0.708
	Senior Radiologist 6	0.832 (119/143)	0.717 (43/60)	0.86 (43/50)	0.817 (76/93)	0.782	0.677
	Junior Radiologist 7	0.790 (113/143)	0.607 (38/56)	0.76 (38/50)	0.806 (75/93)	0.675	0.566
	Junior Radiologist 8	0.727 (104/143)	0.596 (34/57)	0.72 (36/50)	0.795 (74/93)	0.652	0.516
