# Supplementary material for: Development and clinical validation of deep learning for auto-diagnosis of supraspinatus tears
Source: J Orthop Surg Res. 2023 Jun 13;18:426. doi: 10.1186/s13018-023-03909-z (PMC10262398; doi:10.1186/s13018-023-03909-z)
Supplement: Supplementary file 4 — Additional file 4: Figure S3 2-class confusion matrices of models on test set. (A) 2-class confusionmatrices of 2D model on internal test set. (B) 2-class confusion matrices of 3D model on internaltest set. (C) 2-class confusion matrices of 2D model on surgery test set. (D) 2-class confusionmatrices of 3D model on surgery test set. CNN, convolutional neural network; ROC, receiveroperating characteristic. [file 13018_2023_3909_MOESM4_ESM.pdf]

Additional Files 4

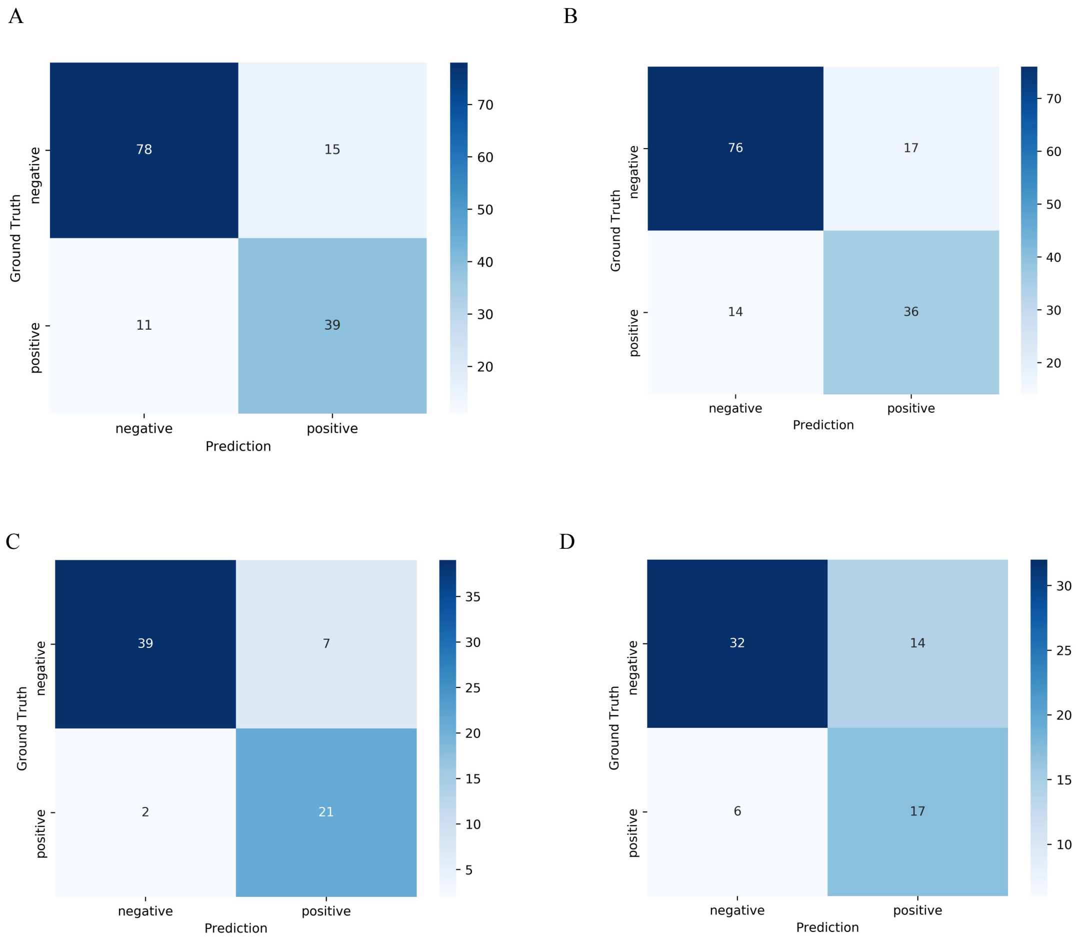

Additional Figure 3. 2-class confusion matrices of models on test set. (A) 2-class confusion matrices of 2D model on internal test set. (B) 2-class confusion matrices of 3D model on internal test set. (C) 2-class confusion matrices of 2D model on surgery test set. (D) 2-class confusion matrices of 3D model on surgery test set. CNN, convolutional neural network; ROC, receiver operating characteristic.
