# Supplementary material for: Development and clinical validation of deep learning for auto-diagnosis of supraspinatus tears
Source: J Orthop Surg Res. 2023 Jun 13;18:426. doi: 10.1186/s13018-023-03909-z (PMC10262398; doi:10.1186/s13018-023-03909-z)
Supplement: Supplementary file 5 — Additional file 5: Table S2 Diagnostic performance of 2D CNN models and reading clinicians on 1.5T and 3.0T MRIexaminations. [file 13018_2023_3909_MOESM5_ESM.pdf]

Additional Files 5

Additional Table 2. Diagnostic performance of 2D CNN models and reading clinicians on 1.5T and 3.0 T MRI examinations.

	Metrics	1.5T	3.0T
Surgery test set	2D CNN	0.848 (0.662-1)	0.957 (0.883-1)
	Senior Surgeon 1	0.8893 (0.8197-0.959)	0.938 (0.8671-1)
	Senior Surgeon 2	0.8355 (0.758-0.9129)	0.8938 (0.802-0.9856)
	Junior Surgeon 3	0.7886 (0.7036-0.8736)	0.8665 (0.7735-0.9595)
	Junior Surgeon 4	0.7702 (0.6769-0.8635)	0.7961 (0.6787-0.9134)
	Senior Radiologist 5	0.7871 (0.6977-0.8765)	0.8402 (0.7353-0.9452)
	Senior Radiologist 6	0.867 (0.7969-0.9371)	0.7876 (0.6657-0.9095)
	Junior Radiologist 7	0.8208 (0.7454-0.8963)	0.8318 (0.7208-0.9427)
	Junior Radiologist 8	0.7395 (0.6432-0.8357)	0.797 (0.6757-0.9182)
Internal test set	2D CNN	0.887 (0.802-0.971)	0.86 (0.749-0.971)
	Senior Surgeon 1	0.8893 (0.8197-0.959)	0.938 (0.8671-1)
	Senior Surgeon 2	0.8355 (0.758-0.9129)	0.8938 (0.802-0.9856)
	Junior Surgeon 3	0.7886 (0.7036-0.8736)	0.8665 (0.7735-0.9595)
	Junior Surgeon 4	0.7702 (0.6769-0.8635)	0.7961 (0.6787-0.9134)
	Senior Radiologist 5	0.7871 (0.6977-0.8765)	0.8402 (0.7353-0.9452)
	Senior Radiologist 6	0.867 (0.7969-0.9371)	0.7876 (0.6657-0.9095)
	Junior Radiologist 7	0.8208 (0.7454-0.8963)	0.8318 (0.7208-0.9427)
	Junior Radiologist 8	0.7395 (0.6432-0.8357)	0.797 (0.6757-0.9182)
